# Designing Smart Space Services by Virtual Reality-Interactive Learning Model on College Entrepreneurship Education

**DOI:** 10.3389/fpsyg.2022.913277

**Published:** 2022-07-22

**Authors:** Yingying Pan

**Affiliations:** Department of Design, Academy of Fine Arts, Henan Normal University, Xinxiang, China

**Keywords:** virtual reality technology, virtual reality interactive learning model, Smart Space, service design, harris corner detection

## Abstract

The purpose was to improve the limitations of traditional entrepreneurship education, realize the virtual interactive learning between college students and teachers, and stimulate students’ exploration of entrepreneurship. This work first discusses the working principle of Virtual Reality (VR) and builds an Interactive Learning Model (ILM) using VR. Then, the VR-ILM is used to design the Smart Space services. Harris Corner Detector (HCD) is used to detect the pixel grayscale change in the Smart Space image window. Further, the VR-ILM-based Smart Space is proposed according to the Smart Space design requirements and principles. Finally, the proposed VR-ILM-based Smart Space is applied to College Entrepreneurship Education (CEE). Its impact on the CEE market, employment in different industries, and students’ satisfaction with CEE are studied. The results show that the proposed VR-ILM-based Smart Space has increased the entrepreneurship teaching courses, entrepreneurship coaching activities, and entrepreneurship practice activities by 4, 6, and 24%, respectively. It has reduced entrepreneurship competitions and other forms of entrepreneurship education by 4 and 16%. The proposed VR-ILM-based Smart Space has dramatically improved the practical teaching of CEE. Meanwhile, real estate services have felt the most significant impact of the proposed VR-ILM-based Smart Space, with an employment increase of 43%. Lastly, students’ satisfaction with entrepreneurship education practice and teaching methods has increased by nearly 50%. The satisfaction with the internal environment has increased to 78%. The satisfaction with the curriculum system, teachers, and industry financing has increased from 30 to 45%, 24 to 36%, and 45 to 63%, respectively. The satisfaction with the teaching goal has increased to 62%. Thus, the proposed VR-ILM-based Smart Space has dramatically improved students’ satisfaction with CEE and has a different impact on the market, industry, and satisfaction with CE. The finding has a certain reference for the VR interactive model.

## Introduction

Higher education in China is entering a stage of high-quality development nowadays. The new journey of socialist modernization must look into contemporary higher education development law to adapt to the development of media integration. Meanwhile, multiple relationships must be handled appropriately to improve colleges’ leadership. The enthusiasm and creativity should be motivated in and out of higher institutions to achieve sustainable higher education in China (Qian et al., 2018; Wu and Song, 2019). In particular, College Entrepreneurship Education (CEE) should learn the experience of foreign universities to cultivate talents’ psychological quality to prepare them for possible innovation and entrepreneurship (Rogoza et al., 2018; Chen, 2019; Wu et al., 2019). In the overall educational ecology in China, CEE is also a critical link to quality education. It can cultivate composite talents, thereby alleviating the employment market, serving economic development, and contributing to social stability (Wu W. et al., 2020). CEE emerged in the United States in 1947, marked by Harvard Business School’s “New Enterprise Management” course. Since then, entrepreneurship education has been pursued worldwide (Wu and Wu, 2017). However, entrepreneurial publicity and education have been emphasized without fruitful and convincing cases. Thus, the positive effect of CEE on students is relatively small. On the other hand, there is no environment for CEE to extend its influence on overall social development (Zheng et al., 2018; Wu and Chen, 2021).

Traditionally, students acquire knowledge passively through text, images, and other multimedia presentations. The learning efficiency is poor. Recently, the barrier-free service design of the Smart Space has been proposed. It emphasizes that the public space must fully consider people’s needs for clothing, food, housing, transportation, and the design of buildings and equipment. Meanwhile, Smart Space should guarantee human safety, convenience, and comfort. Its essence is to return to the scene and participate in its process (Zhang and Alijla, 2019; Geng et al., 2020; Jin et al., 2020; Wu Y. J. et al., 2020). Virtual Reality (VR) technology can visualize entities in multi-dimensional space and enable real-virtual interaction. VR also participates in the evolution process of events according to various means to obtain greater freedom to control and operate the whole environment (D’Cunha et al., 2019; Gao et al., 2019; Tan et al., 2020). VR technology was originated in the National Aeronautics and Space Administration (NASA) Ames laboratory, United States (Barrado-Timón and Hidalgo-Giralt, 2019). Every important milestone in the history of interaction design originates from the collision of technology and human nature. The changes from the initial punching of paper tape to keyboard input, mouse input, and now touch operation, speech recognition, as well as 3D gesture and eye movement recognition have achieved the technological innovation in human-computer interaction (Walmsley and Kersten, 2020). It has been prospected that brain-computer interfaces and mind control will also be implemented. Indeed, every technological innovation and product upgrade will bring about major changes in Human-Computer Interaction (HCI). They all follow a uniform path of conforming to human engineering or ergonomics (Yuan and Wu, 2020).

Based on the above analysis, this work first discusses the working principle of VR and builds an Interactive Learning Model (ILM). Then, the VR-ILM is used to design Smart Space services and uses Harris Corner Detector (HCD) to detect the pixel grayscale change in the Smart Space image window. Consequently, the VR-ILM-based Smart Space is designed according to the Smart Space service design requirements and principles. Finally, the proposed VR-ILM-based Smart Space is applied to CEE. Its impact on different aspects of the market, industry, and students’ satisfaction with CEE is studied. The proposed VR-ILM-based Smart Space has a different impact on the market, industry, and students’ satisfaction with CEE. It has a certain reference for the VR interactive model.

## The Service Design of the Smart Space by the VR-ILM

### The Design of the VR-ILM

**FIGURE 1:**
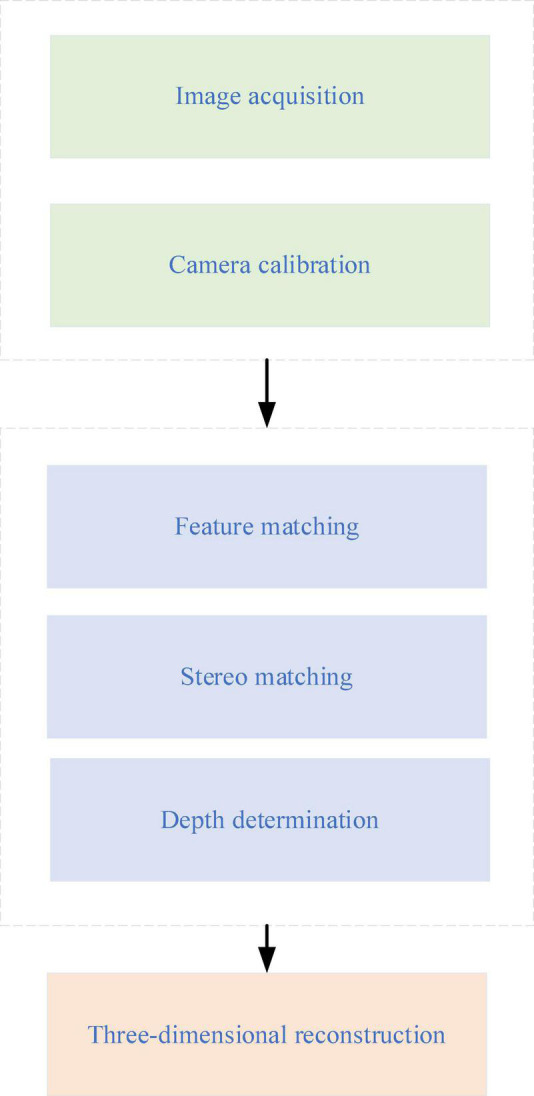
The working principle of Virtual Reality (VR) technology.

Virtual Reality is an emerging cutting-edge technology that involves many fields and has many applications. It combines virtual and reality. Theoretically, VR technology is a computer simulation system that can reconstruct and experience the virtual world. It generates a virtual environment with the computer’s help, immersing visitors in the environment (Flavián et al., 2019). VR technology uses computer equipment to simulate a 3D virtual world, where users can use their sense of vision, hearing, and touch to get an immersive experience. Meanwhile, VR technology has the significant characteristics of multi-sensing, presence, interactivity, and autonomy. Its working principle is shown in Figure 1.

**FIGURE 2:**
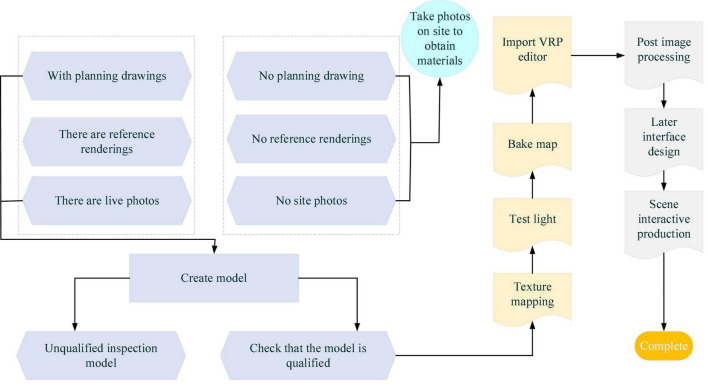
The design process of the Virtual Reality-Interactive Learning Model (VR-ILM).

In Figure 1, the VR system captures real-life images to translate the electronic (digital) signals through various output devices into physical entities, states, or specific relationships. This is where the 3D modeling technique has the most prominent role. Meanwhile, VR is a major direction of simulation technology. It is a collection of simulation and computer graphics, HCI, multimedia, sensing, and network technologies. It is a challenging frontier interdisciplinary subject and research field. In education, VR is mainly used to explain and visualize complex systems and abstract concepts, such as quantum physics. The design process of the VR-ILM is displayed in Figure 2.

**FIGURE 3:**
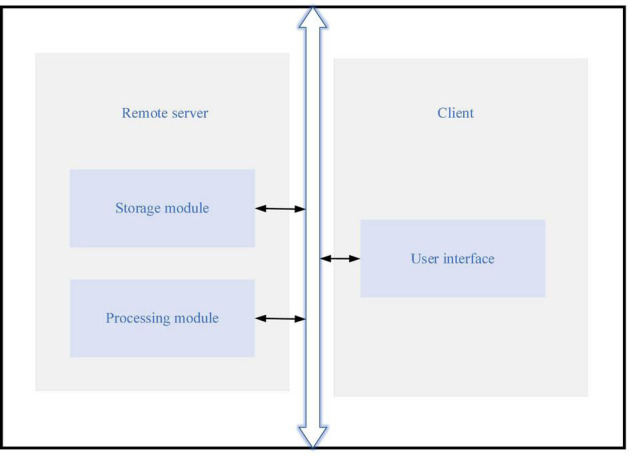
The Virtual Reality-Interactive Learning Model (VR-ILM).

Figure 2 lists the functional modules of a VR-ILM design. The models and texture maps in the virtual scene are derived from the real scene. The texture maps and the plane model of the real scene are collected through the camera, and the Virtual Reality Platform (VRP) editor learns the design of interactive models. According to the design process, a VR-ILM is proposed in Figure 3.

The VR-ILM in Figure 3 includes the client and a remote server terminal. The client terminal is connected with the remote server through a wired/wireless communication network to realize real-time communication. Users can choose a learning scene on the client terminal and access the VR environment. The remote server includes processing and storage modules coupled together: the storage module stores VR simulation data and user attributes. The processing module is configured through the instructions stored in the storage module. The VR simulation data and the previous learning accumulation generate the 3D interactive scene and query structure for the learning request. The client accesses the remote server through the Client/Server (C/S) mode to use the 3D interactive scene. The processing module calls the VR simulation data in the storage module and accumulates previous learning to generate the 3D interactive scene and query structure of the next learning request.

### The Service Design of the Smart Space by the VR-ILM

**FIGURE 4:**
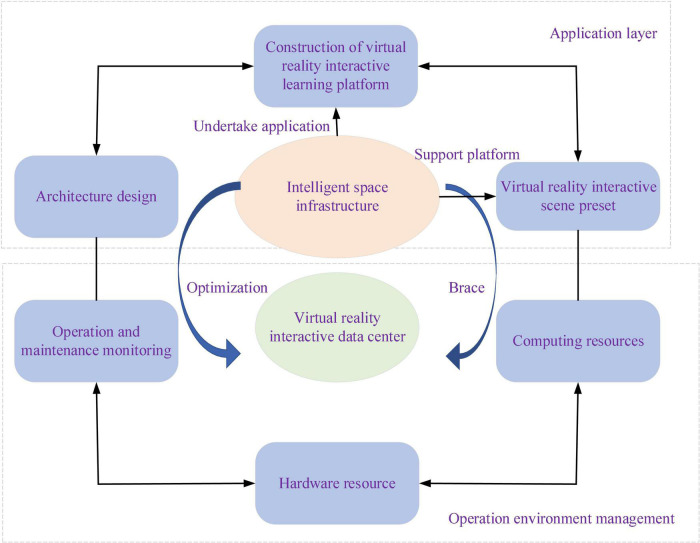
The design framework of Smart Space.

Smart Space is a working and living space embedded with computing, equipment information, and multi-module sensing devices. It has a natural and convenient HCI that provides interactive services for people’s work and life (Lytvyn et al., 2019). The HCI not merely passively executes display and operation commands but actively interacts with people. Thus, it eliminates unnecessary time spent learning the computer commands or operations and saves time waiting by supporting concurrent tasks. Meanwhile, possible overload can be avoided through inter-space resource sharing and distributed processing. The design framework of Smart Space is explained in Figure 4.

In Figure 4, designing the Smart Space solves practical business problems and explores the process from data → information → value by fully utilizing data as resources and Smart Space infrastructure. Environment management is the communication, coordination, and services among Smart Space applications, resources, operating systems, and the network. The principles of service design of Smart Space are listed in Table 1.

**TABLE 1:** The principles of service design of Smart Space.

The principles of service design	Object-oriented	Character
People-oriented	The service affects everyone.	Comprehensive
Cooperation	Stakeholders with different backgrounds and responsibilities	Technical
Iterate	Service design process	Exploratory, dynamic, and experimental
Order	Orderly service design	Visibility
Authenticity	Realistic design object	Value
Comprehensiveness	Whole Service and Whole Business	Sustainability

**FIGURE 5:**
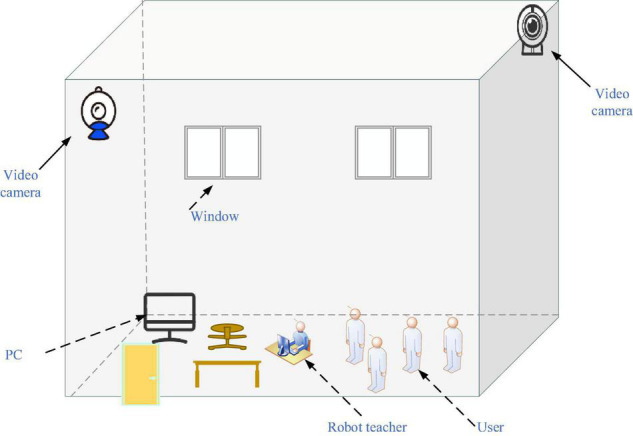
The proposed Virtual Reality-Interactive Learning Model (VR-ILM)-based Smart Space.

In Table 1, the principles of service design are divided into six aspects: people-oriented, cooperation, iteration, order, authenticity, and comprehensiveness (Abdulkareem et al., 2019). According to principles of service design and different audience objects, the VR-ILM-based Smart Space is drawn in Figure 5.

**FIGURE 6:**
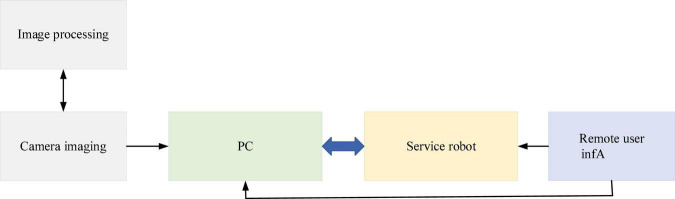
The information transmission process of the proposed Virtual Reality-Interactive Learning Model (VR-ILM)-based Smart Space.

Figure 5 is a typical multi-user interactive working-learning environment of Smart Space. It can distinguish the robot teacher and virtual users and monitor user activities. Here, personal computer (PC) is the main server and is mainly responsible for the information processing, storage, and simultaneous interaction. Studies have shown that some students can feel worried and embarrassed in CEE by not being able to interact. The proposed VR-ILM-based Smart Space can mitigate their worry and embarrassment through immersive practice and entrepreneurial activities. It allows students to review the course multiple times until they fully comprehend it and encourage them to socialize better by responding to and sharing in the same virtual world. Further, interactive learning in virtual space enables students to form unique learning cognition and realistic and timely teacher-student interactions. The information transmission process of the service design of Smart Space is illustrated in Figure 6.

In Figure 6, the proposed VR-ILM-based Smart Space enhances the real space through VR-ILM. The service design is based on camera-captured images. The captured Composite Video Signal (CVS) must be digitized before the subsequent video processing. Secondly, according to the information transmission between the PC and the service robot, the speed transmission requirements of the 3D information acquisition system, the Programmable Communication Interface (PCI), is used to complete the information exchange between the service robot of smart space and the general PC (Podpora et al., 2020). The feature recognition algorithm is selected for image processing. Objects in the Smart Space are distinguished through image features. The gradient of the image corner changes much. Thus, the focus can be used as a basis for judgment. HCD has partial invariance and rotation invariance for affine change of image gray, which ensures that the image eigenvalues remain unchanged. Thus, the pixel grayscale change in the window is detected using HCD. Sliding in any direction and a large grayscale change (Yang, 2019) are the indication of corners in the window. When the window slides by [*u*,*v*], the grayscale changes of the corresponding pixels in the window before and after sliding are calculated as:


(1) $$E\,\left(u,v\right)=\sum_{x,y}w\,\left(x,y\right)\,{\left[I\,\left(x+u,y+v\right)-I\,\left(x,y\right)\right]}^{2}$$

In Eq. (1), [*u*, *v*] is the offset of the window. (*x*, *y*) is the corresponding position of pixel coordinate in the window. There are as many positions as the window size is. *I* is the intensity coefficient. *w* (*x*, *y*) is the weight coefficient of the window. The simplest case is that the weight coefficient *w* of all pixels in the window is 1. Sometimes, the *w* (*x*, *y*) function is set to a bivariate normal distribution with the center of the window as the origin. If the center point of the window is a corner, the grayscale change of this point should be the most severe before and after sliding. Thus, the weight coefficient of this point can be set larger, which means that when the window slides, this point contributes more to the grayscale change. The grayscale changes of points farther from the center of the window are almost gentle. The weight coefficient of these points can be set smaller to indicate that the point contributes less to the grayscale change. According to the above expression, when the window slides on a flat area, it is conceivable that the grayscale will not change. Then, *E* (*u*, *v*) = 0. *E* (*u*, *v*) can be updated according to the Taylor formula, and the Taylor expansion is shown in Eq. (2).


(2) f(x)=f(x0)+f(x0)′(x-x0) +⋯+f(n⁢1)⁢(x0+θ⁢(x-x0))(n+1)!⁢(x-x0)n+1

In Eq. (2), 0 < θ < 1, the function f(x) has a derivative up to the *n+1* order in the open interval (a, b) containing x. Then, when the function is in this interval, it can be expanded to about (*x*−*x*_0_), the sum of the polynomial and remainder. *E* (*u*, *v*) can be updated according to the Taylor formula, as shown in Eq. (3).


(3) $$E\,\left(u,v\right)\approx \left[u,v\right]\,M\,\left[\begin{array}{c} u\\ v \end{array}\right]$$

In Eq. (3), M is the autocorrelation matrix calculated by each pixel, and the specific mathematical expression is shown in Eq. (4). $\left[\begin{array}{ccc} I_{x}^{2} & & I_{x}\,I_{y}\\ I_{x}\,I_{y} & & I_{y}^{2} \end{array}\right]$ denotes the product of the gradient *I*_*x*_ and *I*_*y*_ components.


(4) $$M=\sum_{x,y}w\,\left(x,y\right)\,\left[\begin{array}{ccc} I_{x}^{2} & & I_{x}\,I_{y}\\ I_{x}\,I_{y} & & I_{y}^{2} \end{array}\right]$$

In Eq. (4), statistical analysis is performed on the gradient in the *x*-direction and the gradient in the *y*-direction of each pixel in the window. *I*_*x*_ and *I*_*y*_ are the coordinate axes, so the gradient coordinates of each pixel can be expressed as (*I*_*x*_, *I*_*y*_). The measure of the response of each corner is shown in Eq. (5).


(5) $$R=d\,e\,t\,M-k\,{\left(t\,r\,a\,c\,e\,M\right)}^{2}$$

In Eq. (5), R is a function. k is a constant, and the general value is 0.04–0.06. These parameters can only adjust the shape of the function. Finally, the threshold of R is set to judge the corner.

## Results and Discussion

Currently, CEE has developed from the stage of trial and exploration to a new stage of refined, hierarchical, and diversified development. Nevertheless, it also faces some problems. For example, in the integration of production and education, there is a phenomenon of “government policies gaining negative social feedback” and a situation of “active college efforts gaining negative enterprise feedback” in school-enterprise cooperation. CEE has shown a disciplinary tendency, and schools and teachers have a minimal understanding of CEE. Thus, to achieve high-level national entrepreneurship education, the primary “employment education” stage must be abandoned to integrate CEE into the talent training process. The following experiment will discuss the impact of the service design of Smart Space of the VR-ILM on CEE. The experiment focused on undergraduates from China University of Mining and Technology, such as students studying at school, students starting businesses, unemployed students, students with fixed jobs, and some masters and doctoral students. Here, the WeChat platform was used to send Questionnaire Survey (QS) to explore the impact of the proposed VR-ILM-based Smart Space on CEE. Altogether, 112 QSs were collected, and 104 valid ones were recovered, with an effective recovery rate of 92.86%. Then, the influencing factors of CEE were analyzed by investigating students with different entrepreneurship situations. Table 2 specifies the QS results and analysis.

**TABLE 2:** Analysis of the influencing factors of College Entrepreneurship Education (CEE) on students with different entrepreneurship situations.

Influencing factors of CEE	Studying in school	Starting a business	Unemployed	Fixed job
Weak market awareness	48.4%	48.7%	44.6%	46.1%
Employment pressure	56.3%	69.2%	66.0%	3.9%
Policy	21.6%	22.6%	24.3%	18.7%
Lack of educational concepts	44.6%	46.2%	38.0%	32.5%
Incomplete education system	26.1%	47.3%	49.9%	26.7%
Faculty	15.4%	68.3%	57.8%	10.7%
Entrepreneurship education environment	23.4%	48.7%	63.9%	23.4%
Funds	23.8.0%	41.2%	49.6%	15.6%

According to Table 2, the influential factors of CEE on students mainly include weak market awareness, employment pressure, and lack of entrepreneurship educational concepts. Then, the VR-ILM-based Smart Space’s impact was analyzed on the CEE market, the employment in different industries, and students’ satisfaction with CEE.

### Impact of VR-ILM-Based Smart Space on the College Entrepreneurship Education Market

**FIGURE 7:**
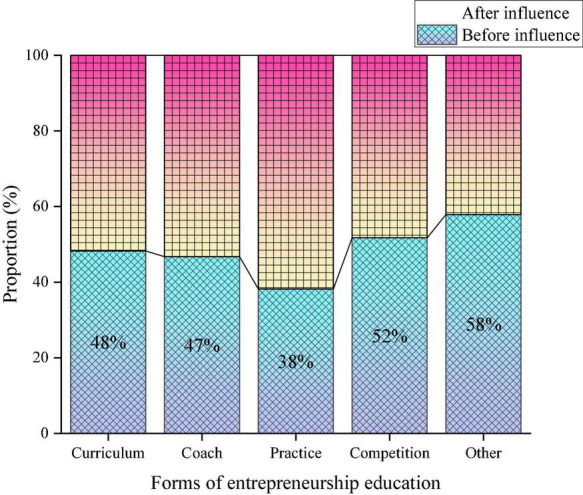
The proposed Virtual Reality-Interactive Learning Model (VR-ILM)-based Smart Space’s influence on the form of College Entrepreneurship Education (CEE).

According to the development of contemporary entrepreneurship education, CEE forms are classified into entrepreneurship teaching courses, entrepreneurship coaching activities, entrepreneurship practice activities, entrepreneurship competition activities, and other activities. The influence of the proposed VR-ILM-based Smart Space on the form of CEE is outlined in Figure 7.

In Figure 7, after introducing the VR-ILM-based Smart Space, entrepreneurship teaching courses, entrepreneurship coaching activities, and entrepreneurship practice activities are increased by 4, 6, and 24%, respectively. Nevertheless, there has been a certain decline in entrepreneurship competitions and other forms of entrepreneurship education, by 4 and 16%, respectively. It means that the proposed VR-ILM-based Smart Space has greatly improved the practical teaching of CEE.

### Impact of VR-ILM-Based Smart Space on Employment in Different Industries

**FIGURE 8:**
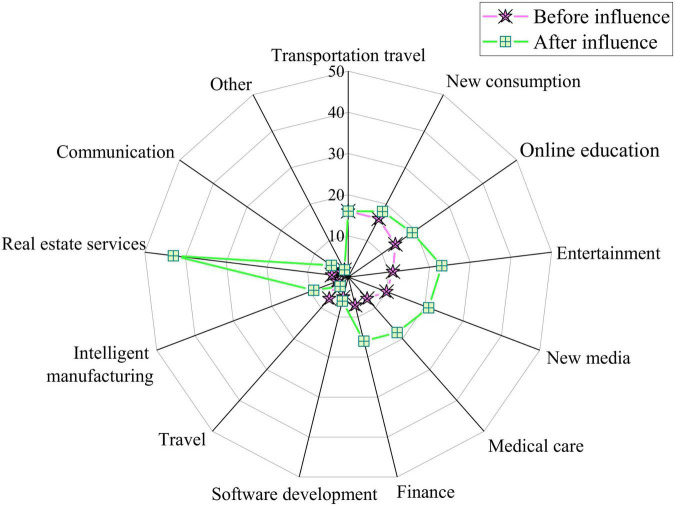
The impact of the proposed Virtual Reality-Interactive Learning Model (VR-ILM)-based Smart Space on employment in different industries.

The impact of the proposed VR-ILM-based Smart Space on employment in different industries was analyzed in colleges from 2019 to 2021. Industries are classified into 13 forms: transportation, new consumption, online education, entertainment, new media, medical care, finance, software development, travel, intelligent manufacturing, real estate services, communications, and others. Figure 8 plots the impact of the proposed VR-ILM-based Smart Space on employment in different industries.

Figure 8 indicates that introducing VR-ILM-based Smart Space has helped to increase students’ employment in different industries. For instance, real estate services have felt the largest impact, with an employment increase of 43%, followed by the entertainment industry (increased by 23%). Meanwhile, employment in the new media, medical, and financial industries has increased by 21, 18, and 18%, respectively. Employment in other industries has also increased by a small margin. Thus, the proposed VR-ILM-based Smart Space greatly impacts the development of real estate services through CEE.

### Impact of VR-ILM-Based Smart Space on Student’s Satisfaction With College Entrepreneurship Education

**FIGURE 9:**
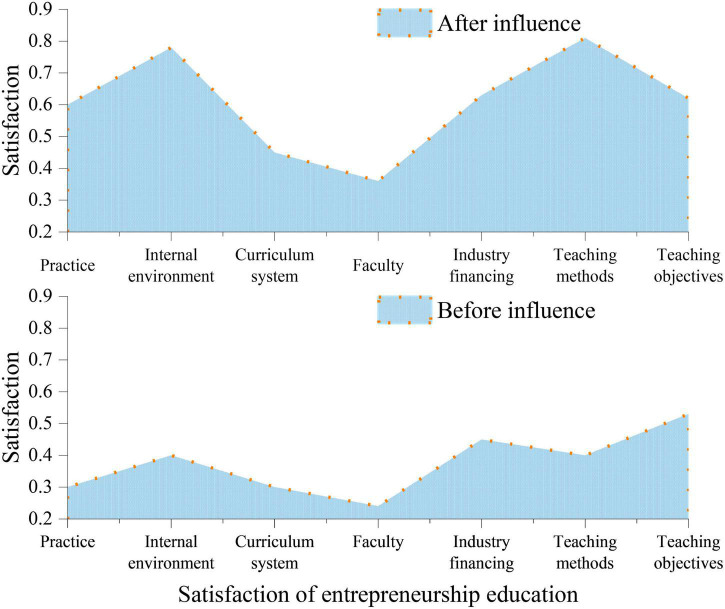
Analysis of the impact of the proposed Virtual Reality-Interactive Learning Model (VR-ILM)-based Smart Space on students’ satisfaction with College Entrepreneurship Education (CEE).

Here, it considered the practice of entrepreneurship education in colleges, internal environment, curriculum system, faculty, industry financing, teaching methods, and teaching objectives. Figure 9 shows the impact of the proposed VR-ILM-based Smart Space on students’ satisfaction with CEE.

According to Figure 9, after introducing the proposed VR-ILM-based Smart Space, students’ satisfaction with all aspects of CEE has been dramatically improved. Specifically, the satisfaction with entrepreneurship education practice and teaching methods has increased by nearly 50%. The satisfaction with the internal environment has increased to 78%. The satisfaction with the curriculum system has increased from 30 to 45%, and the satisfaction with teachers has increased from 24 to 36%. The satisfaction with the financing has increased from 45 to 63%, and the satisfaction with the teaching objectives has increased to 62%.

## Conclusion

According to VR technology, the ILM is built and used for the Smart Space service design. Consequently, a VR-ILM-based Smart Space is proposed. Its impact on CEE is studied by recruiting students of the China University of Mining and Technology as the research subjects and investigating them by the QS method. The results show that after introducing the VR-ILM-based Smart Space, entrepreneurship teaching courses, entrepreneurship coaching activities, and entrepreneurship practice activities have increased by 4, 6, and 14%, respectively. By comparison, entrepreneurship competition activities and other forms of entrepreneurship education have decreased by 4 and 16%, respectively. Under the influence of the VR-ILM-based Smart Space, students’ employment in different industries has grown sustainably. Real estate services have felt the largest impact, with a 43% increase, followed by the entertainment industry (an increase of 23%). Employment in the new media, medical, and financial industries has increased by 21, 18, and 18%, respectively. Other industries also have shown slight increases in employment. Meanwhile, students’ satisfaction with CEE has been greatly improved: satisfaction with entrepreneurship education practice and teaching methods and internal environment has increased by nearly 50 and 78%, respectively. Their satisfaction with the curriculum system, teachers, and industry financing has increased from 30 to 45%, 24 to 36%, and 45 to 63%, respectively. The satisfaction with teaching goals has increased to 62%. The proposed VR-ILM-based Smart Space has a different influence on the CEE market, industrial employment, and satisfaction with CEE. It has a certain reference to design the VR interactive model. Last but not least, CEE is transforming every day. Thus, it is necessary to continue researching CEE following the technological development of the times. Future work is expected to consider more interactive intentions and interactive scenes of learners in the VR-ILM-based Smart Space. Additionally, to cope with post-COVID economic recovery and make up for the traditional education, expanding the VR-ILM-based Smart Space experience group is the development direction of future research.

## Data Availability Statement

The raw data supporting the conclusions of this article will be made available by the authors, without undue reservation.

## Ethics Statement

The studies involving human participants were reviewed and approved by the Henan Normal University Ethics Committee. The patients/participants provided their written informed consent to participate in this study. Written informed consent was obtained from the individual(s) for the publication of any potentially identifiable images or data included in this article.

## Author Contributions

The author confirms being the sole contributor of this work and has approved it for publication.

## Conflict of Interest

The author declares that the research was conducted in the absence of any commercial or financial relationships that could be construed as a potential conflict of interest.

## Publisher’s Note

All claims expressed in this article are solely those of the authors and do not necessarily represent those of their affiliated organizations, or those of the publisher, the editors and the reviewers. Any product that may be evaluated in this article, or claim that may be made by its manufacturer, is not guaranteed or endorsed by the publisher.

## References

[B1] Abdulkareem S. A. Mustafa Y. T. Augustijn E. W. GeoInformatica T. F. (2019). Bayesian networks for spatial learning: a workflow on using limited survey data for intelligent learning in spatial agent-based models. *Geoinformatica* 23 243–268. 10.1007/s10707-019-00347-0

[B2] Barrado-Timón D. A. Hidalgo-Giralt C. (2019). The historic city, its transmission and perception via augmented reality and virtual reality and the use of the past as a resource for the present: a new era for urban cultural heritage and tourism? *Sustainability* 11:2835. 10.3390/su11102835

[B3] Chen M. (2019). The impact of expatriates’ cross-cultural adjustment on work stress and job involvement in the high-tech industry. *Front. Psychol.* 10:2228. 10.3389/fpsyg.2019.02228 31649581 PMC6794360

[B4] D’Cunha N. M. Nguyen D. Naumovski N. McKune A. J. Kellett J. Georgousopoulou E. N. (2019). A mini-review of virtual reality-based interventions to promote well-being for people living with dementia and mild cognitive impairment. *Gerontology* 65 430–440. 10.1159/000500040 31108489

[B5] Flavián C. Ibáñez-Sánchez S. Orús C. (2019). Integrating virtual reality devices into the body: effects of technological embodiment on customer engagement and behavioral intentions toward the destination. *J. Travel Tour. Mark.* 36 847–863. 10.1080/10548408.2019.1618781

[B6] Gao T. Zhang T. Zhu L. Gao Y. Qiu L. (2019). Exploring psychophysiological restoration and individual preference in the different environments based on virtual reality. *Int. J. Env. Res. Public Health* 16:3102. 10.3390/ijerph16173102 31455015 PMC6747099

[B7] Geng Z. Chen N. Han Y. Ma B. (2020). An improved intelligent early warning method based on MWSPCA and its application in complex chemical processes. *Can. J. Chem. Eng.* 98 1307–1318. 10.1002/cjce.23674

[B8] Jin Z. Han Q. Zhang K. (2020). An intelligent fault diagnosis method of rolling bearings based on Welch power spectrum transformation with radial basis function neural network. *J. Vib. Control.* 26 629–642. 10.1177/1077546319889859

[B9] Lytvyn V. Vysotska V. Demchuk A. Demkiv I. Ukhanska O. Hladun V. (2019). Design of the architecture of an intelligent system for distributing commercial content in the internet space based on SEO-technologies, neural networks, and machine learning. Восточно-Европейский журнал передовых технологий 2 15–34. 10.15587/1729-4061.2019.164441

[B10] Podpora M. Gardecki A. Beniak R. Klin B. Vicario J. L. Kawala-Sterniuk A. (2020). Human interaction smart subsystem—extending speech-based human-robot interaction systems with an implementation of external smart sensors. *Sensors* 20:2376. 10.3390/s20082376 32331291 PMC7219337

[B11] Qian J. Song B. Jin Z. Wang B. Chen H. (2018). Linking empowering leadership to task performance, taking charge, and voice: the mediating role of feedback-seeking. *Front. Psychol.* 9:2025. 10.3389/fpsyg.2018.02025 30410461 PMC6209672

[B12] Rogoza R. Żemojtel-Piotrowska M. Kwiatkowska M. M. Kwiatkowska K. (2018). The bright, the dark, and the blue face of narcissism: the Spectrum of Narcissism in its relations to the metatraits of personality, self-esteem, and the nomological network of shyness, loneliness, and empathy. *Front. Psychol.* 9:343. 10.3389/fpsyg.2018.00343 29593627 PMC5861199

[B13] Tan Y. Niu C. Zhang J. (2020). Head-mounted, display-based immersive virtual reality marine-engine training system: a fully immersive and interactive virtual reality environment. *IEEE Syst. Man Cybern. Mag.* 6 46–51. 10.1109/MSMC.2019.2948654

[B14] Walmsley A. P. Kersten T. P. (2020). The IMPERIAL Cathedral in Königslutter (Germany) as an immersive experience in virtual reality with integrated 360 panoramic photography. *Appl. Sci.* 10:1517. 10.3390/app10041517

[B15] Wu W. Wang H. Wu Y. J. (2020). Internal and external networks, and incubatees’ performance in dynamic environments: entrepreneurial learning’s mediating effect. *J. Technol. Transf.* 46 1707–1733. 10.1007/s10961-020-09790-w

[B16] Wu W. Wang H. Zheng C. Wu Y. J. (2019). Effect of narcissism, psychopathy, and machiavellianism on entrepreneurial intention—the mediating of entrepreneurial self-efficacy. *Front. Psychol.* 10:360. 10.3389/fpsyg.2019.00360 30846958 PMC6393355

[B17] Wu Y. J. Chen J. C. (2021). Stimulating innovation with an innovative curriculum: a curriculum design for a course on new product development. *Int. J. Manag. Educ.* 19:100561. 10.1016/j.ijme.2021.100561

[B18] Wu Y. J. Liu W. J. Yuan C. H. (2020). A mobile-based barrier-free service transportation platform for people with disabilities. *Comput. Hum. Behav.* 107:105776. 10.1016/j.chb.2018.11.005

[B19] Wu Y. Song D. (2019). Gratifications for social media use in entrepreneurship courses: learners’ perspective. *Front. Psychol.* 10:1270. 10.3389/fpsyg.2019.01270 31214081 PMC6555126

[B20] Wu Y. Wu T. A. (2017). A decade of entrepreneurship education in the Asia Pacific for future directions in theory and practice. *Manag. Decis.* 55 1333–1350.

[B21] Yang X. (2019). The method of removing drift signal from rice remote sensing image in ecological environment dynamic monitoring. *Ekoloji* 28 1673–1678.

[B22] Yuan C. H. Wu Y. J. (2020). Mobile instant messaging or face-to-face? Group interactions in cooperative simulations. *Comput. Hum. Behav.* 113:106508. 10.1016/j.chb.2020.106508

[B23] Zhang X. Alijla B. (2019). Research on inheritance and innovation mode of erhu art development based on intelligent algorithm. *J. Intell. Fuzzy Syst.* 37 3327–3334. 10.3233/JIFS-179135

[B24] Zheng W. Wu Y. Chen L. (2018). Business intelligence for patient-centeredness: a systematic review. *Telemat. Inform.* 35 665–676. 10.1016/j.tele.2017.06.015

